# Effects of medium-chain fatty acids as alternatives to ZnO or antibiotics in nursery pig diets

**DOI:** 10.1093/tas/txaa151

**Published:** 2020-08-12

**Authors:** Payton L Dahmer, Grace E Leubcke, Annie B Lerner, Cassandra K Jones

**Affiliations:** Department of Animal Sciences & Industry, Kansas State University, Manhattan, KS

**Keywords:** antibiotic replacement, feed efficiency, MCFA, nursery pig

## Abstract

The objective of this experiment was to evaluate the effects of medium-chain fatty acids (MCFA) on nursery pig performance in place of ZnO and carbadox. In this trial, 360 weanling pigs (DNA 200 × 400; 5.4 ± 0.07 kg BW) were fed for 35 d, with 6 pigs/pen and 10 replicate pens/treatment. Upon weaning, pigs were weighed and allotted to pens based on BW in a completely randomized design to one of six treatment diets: 1) Negative control (no added ZnO or carbadox); 2) Control + 3,000 ppm ZnO in phase 1 and 2,000 ppm ZnO in phase 2; 3) Control + 50 g/ton carbadox; 4) Control + C6:C8:C10 MCFA blend; 5) Control + Proprietary Oil Blend (Feed Energy Corp.); and 6) Control + monolaurate blend (FORMI GML from ADDCON). Treatment diets were fed through two dietary phases and a common diet fed through phase three. Pigs and feeders were individually weighed on a weekly basis to determine average daily gain (ADG) and average daily feed intake (ADFI). From days 0 to 19, pigs being fed the ZnO or Carbadox diets had the greatest ADG. These pigs had significantly higher (*P* < 0.05) ADG than pigs fed the control or Feed Energy Proprietary Oil Blend, whereas pigs fed the C6:C8:C10 blend or FORMI GML diets had similar (*P* > 0.05) ADG compared with those fed carbadox. These effects were primarily driven by feed intake, which was greatest (*P* < 0.05) in pigs fed ZnO and carbadox. Treatment diet had a marginally significant effect (*P* = 0.078) on G:F. Increased day 19 BW (*P* < 0.05) was observed for pigs fed ZnO and carbadox compared with the negative control, whereas other treatments were intermediate. Additionally, blood data and fecal scores were collected throughout the trial. On day 21, pigs fed ZnO or carbadox had higher (*P* < 0.0001) glucose values than those fed the Feed Energy Proprietary Oil Blend, with other diets being intermediate, showing potential health benefits of carbadox. Although ZnO resulted in higher glucose values, it may also contribute to hepatic issues. Although replacing ZnO and carbadox with MCFA did not result in significant changes in gut microflora, it did affect fecal consistency by softening the feces during the treatment period. Overall, these results show that ZnO and carbadox are valuable additives to help maximize growth performance in early stages of the nursery. Some MCFA products, like FORMI GML, may result in similar performance, whereas others restrict it. Thus, additional research is needed to study the effectiveness of MCFA to replace ZnO or feed-based antibiotics.

## INTRODUCTION

The postweaning period is typically a time of health challenge and limited growth performance. Pigs can be stressed from being placed in a new environment, and immature digestive systems can result in reduced feed intake and feed efficiency. Additionally, increased risk for intestinal health problems can often be prevalent with diarrhea stemming from bacterial sources (Pluske, 2013). Antimicrobial agents have been utilized for decades to treat these conditions and ultimately improve nursery pig health and growth performance. For example, supplementation of pharmacological levels (2,000 to 3,000 ppm) of ZnO is a common practice to reduce postweaning diarrhea (Liu et al., 2018). Additionally, feed-based antibiotics, such as carbadox, are widely used additives in swine diets, especially during the nursery stage when newly weaned pigs are subject to enteric diseases and reduced feed intake. Controlled research has shown that including antibiotic growth promoters, like carbadox, can increase growth rate and feed efficiency in weanling pigs by 16.4% and 6.9%, respectively (Cromwell, 2002). Despite these benefits, concerns with potential antibiotic resistance and antibiotic residue in animal products have surfaced (Bager et al., 2000; Gallois et al., 2009). Additionally, the use of pharmacological levels of ZnO has posed environmental concerns due to increased excretion of zinc in swine waste utilized as fertilizer (Jondreville et al., 2003). That said, their use is strictly regulated by the FDA to avoid the risk of potential residues and to maintain environmental and consumer health. With these regulations increasing and a rise in consumer pressure to eliminate the use of feed-based antibiotics in swine production, this leaves swine producers searching for antimicrobial replacements that can yield the same positive outcomes, while avoiding any negative consequences ( Landers et al., 2012;Center for Disease Control, 2013). One potential alternative is thought to be medium-chain fatty acids (MCFA). MCFA are saturated fatty acids with carbon chains 6 to 12 atoms long and consist of caproic (C6), caprylic (C8), capric (C10), and lauric (C12) acids that naturally occur in triglycerides of various feed ingredients. Their ability to be easily digested allows them to be utilized by the pig for growth, or by cells within the pig’s gut to improve development and overall health (Zentek et al., 2011). Since MCFA are directly absorbed into circulation and easily oxidized by the liver, they can also serve as a very rapid energy source for pigs during stressful times (Babayan, 1987; Lee et al., 1994). Their inclusion in swine diets has been demonstrated to reduce the risks of viruses in swine feed, and Cochrane et al. (2018) described their ability to replace 400 g/ton chlortetracycline in phase 2 nursery diets. Additionally, controlled research has reported increased growth performance when MCFA are fed in mid-to-late-nursery diets, even in the absence of health challenges (Thomson et al., 2018; Thomas et al., 2018). However, field research has shown mixed results, especially when feeding begins in early nursery. Therefore, the objective of this study was to evaluate the effectiveness of three different MCFA combinations as replacements for ZnO and carbadox on growth performance, fecal consistency, fecal dry matter, and blood parameters during the nursery phase.

## MATERIALS AND METHODS

All experimental procedures adhered to guidelines for the ethical and humane use of animals for research according to the Guide for the Care and Use of Agricultural Animals in Research and Teaching (FASS, 2010) and were approved by the Institutional Animal Care and Use Committee at Kansas State University (IACUC #4036.20).

### Animal Housing, Dietary Treatments, and Experimental Design

**Figure 1.**
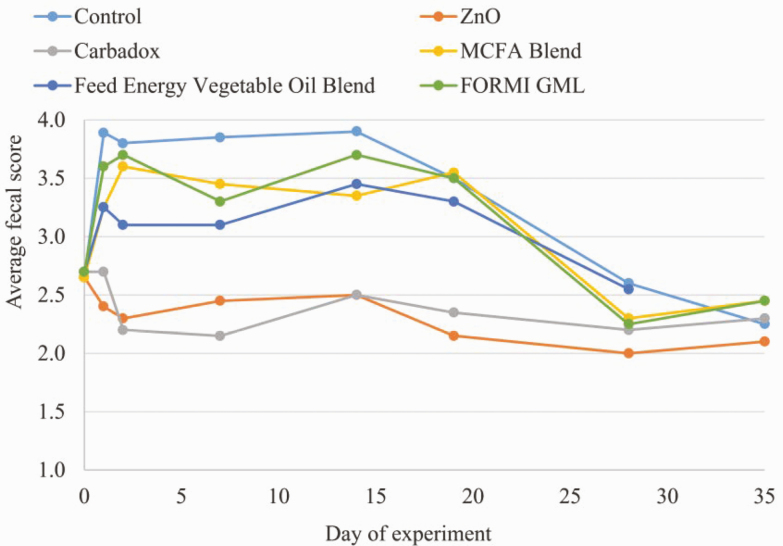
Impact of dietary treatment on fecal score. Fecal scoring was categorized as a numerical scale from 1 to 5 as follows: 1, hard pellet-like feces; 2, firm formed stool; 3, soft moist stool that retains shape; 4, soft unformed; and 5, watery liquid stool.

A total of 360 weanling pigs (DNA 200 × 400; 5.4 ± 0.07 kg BW; approximately 21 d of age) were used in a 35-d experiment with 6 pigs per pen and 10 replicate pens per treatment. Upon weaning, pigs were individually weighed and allotted to pens based on BW to one of six dietary treatments: 1) Negative control (no added ZnO or carbadox); 2) Control + 3,000 ppm ZnO in phase 1 and 2,000 ppm ZnO in phase 2; 3) Control + 50 g/ton carbadox; 4) Control + C6:C8:C10 MCFA blend; 5) Control + Proprietary Oil Blend (Feed Energy Corp.); and 6) Control + monolaurate blend (FORMI GML from ADDCON). Diets were isocaloric, with choice white grease used to balance the energy level. Diets were fed in three phases: phase 1 from days 0 to 7; phase 2 from days 7 to 21; and phase 3 from days 21 to 35 (Figure 1). Phase 3 was a common diet fed to all pigs. All diets were made at the O.H. Kruse Feed Mill (Kansas State University, Manhattan, KS) and were fed in pellet form in phase 1 and in meal form in phases 2 and 3 of the nursery. Diets were also blinded and analyzed for proximate analysis and fatty acid profile at Midwest Laboratories (Midwest Laboratories, Omaha, NE). Target conditioning temperature for pelleting was ~51.7 °C for 30 s, with target hot pellet temperature ~71.1 °C. Pelleting parameters were die size of 3/16″ x 1 1/4 ″ (L/D = 6.0), 1.560 lb/h production rates, and approximately 72 °F ambient temperature.

Pigs were housed in a controlled environment nursery facility (Kansas State University Swine Research and Teaching Center, Manhattan, Kansas) with six pigs per pen. Each pen (1.52 × 1.52 m) included a 4-hole dry self-feeder and a cup drinker to provide all pigs ab libitum access to feed and water.

### Sample Collection, Analyses, and Calculations

All pigs and feeders were weighed on a weekly basis to determine average daily gain (ADG) and average daily feed intake (ADFI). Whole blood samples were collected on days 0 and 21 and submitted to the Kansas State University Veterinary Diagnostic Laboratory (Kansas State University, Manhattan, KS) for complete blood panel, serum chemistry, and hepatic profile. Additionally, fecal swabs were taken from the same three pigs in each pen on days 0, 7, 14, 21, 28, and 35. Three fecal samples from the same pen were pooled for subsequent analysis for fecal microflora and antimicrobial resistance. Fecal scoring was conducted by two independent, trained scorers on days 0, 1, 2, 7, 14, 19, 28, and 35 to categorize the consistency of piglet feces per litter. A numerical scale from 1 to 5 was used: 1, being hard pellet-like feces; 2, a firm formed stool; 3, a soft moist stool that retains shape; 4, a soft unformed; and 5, a watery liquid stool.

Chemical composition of feed samples were analyzed at Midwest Laboratories (Midwest Laboratories, Omaha, NE). Assays included DM using a drying oven (method 930.15; AOAC, 2007), crude protein (CP) as N × 6.25 using the combustion method (Nitrogen Determinator; model TruMac N, Leco Corporation, St. Joseph, MI; method 990.03; AOAC, 2007) and total phosphorous (method 985.01; AOAC, 2007). Additionally, diets were analyzed for fatty acid profiles to determine the levels of C6:0, C8:0, C:10, and C:12 fatty acids (method 996.06, AOAC, 2007).

Data were analyzed using the GLIMMIX procedure of SAS (SAS Institute, Inc., Cary, NC) with pen as the experimental unit and room as a random effect. Results were considered significant if *P* ≤ 0.05 and marginally significant if 0.05 > *P* ≤ 0.10.

## RESULTS AND DISCUSSION

### Nursery Pig Growth Performance

In the first week postweaning, pigs fed diets containing carbadox had greater (*P* < 0.05) ADG than those fed the MCFA or Feed Energy Proprietary Oil Blend. Feed intake was greater (*P* < 0.05) when pigs were fed diets supplemented with ZnO compared with those with the MCFA or Feed Energy Proprietary Oil Blend. This led to a marginally significant impact of diet on G/F from days 0 to 7, with the greatest feed efficiency occurring in pigs fed carbadox or FORMI GML and the poorest feed efficiency in pigs fed the MCFA blend. Although the FORMI GML product resulted in similar performance as ZnO and carbadox, the other MCFA products had adverse impacts. These findings somewhat refute research done by Hong et al. (2012) that describes the ability of MCFA to increase ADFI for the first two weeks following weaning when compared with diets containing antibiotics. Similarly, Rodas et al. (1990) found that MCFA inclusion at a rate of 20 to 60 g/kg could increase ADG and G/F in weanling pigs shortly after supplementation. A primary reason to describe this is the piglet’s ability to effectively absorb and use MCFA (Odle et al., 1989). More specifically, Odle et al. (1999) explain that MCFA are able to be absorbed without hydrolysis by lipase, and they enter the liver faster; thus, they are hydrolyzed quicker and digested easier. However, the discrepancies between results of these experiments and the current study warrant further research to evaluate MCFA impact on feed intake and feed efficiency during the first week postweaning.

In phase 2 (days 7 to 19), pigs fed diets containing ZnO had greater (*P* < 0.05) ADG than those fed either the control or diets containing the MCFA or Feed Energy Proprietary Oil Blends. This was due to pigs consuming the ZnO diet having greater (*P* < 0.05) feed intake than those fed the MCFA or Feed Energy Proprietary Oil Blends and greater (*P* < 0.05) G/F than pigs consuming the control diet. Similarly, Cemin et al. (2018) showed that weanling pigs being fed added ZnO had increased ADFI and enhanced growth performance. Yet, research conducted byMellick et al. (2019) showed increased ADFI by inclusion of a MCFA blend. This contrasts results found in the current study, suggesting that further evaluation of feeding MCFA during mid-nursery is needed to better understand how different fatty acid blends and commercial products can affect growth performance (Table 1).

**Table 1.** Diet composition (as-fed basis)

	Phase 1	Phase 2	Phase 3
Ingredient, %			
Corn	44.8	56.7	65.6
Soybean meal, 46.5% CP	18.1	29.1	30.2
Fish meal	4.5	–	–
Spray dried whey	25.0	10.0	–
Monocalcium phosphate, 21% P	0.30	0.90	0.95
Limestone	0.25	0.98	1.00
Sodium chloride	0.30	0.55	0.58
l-Lysine	0.43	0.50	0.55
dl-Methionine	0.23	0.21	0.23
l-Threonine	0.21	0.24	0.25
l-Tryptophan	0.06	0.04	0.07
l-Valine	0.11	0.11	0.14
Trace mineral premix^*a*^	0.15	0.15	0.15
Vitamin premix^*b*^	0.25	0.25	0.25
Phytase^*c*^	0.08	0.08	0.08
Hamlet Protein 300^*d*^	3.75	–	–
Feed Additive^*e*^	Varied	Varied	n/a
Total	100.0	100.0	100.0
Calculated analysis			
Standardized ileal digestibility (SID) amino acids, %			
Lys	1.40	1.35	1.24
Ile:Lys	56	55	57
Lue:Lys	109	112	119
Met:Lys	38	36	36
Met & Cys:Lys	58	57	58
Thr:Lys	65	65	65
Trp:Lys	20.3	19.1	18.6
Val:Lys	68	67	67
ME, Mcal/kg	3.42	3.28	3.42
CP, %	21.0	20.6	20.0
SID Lys:ME, g/Mcal	5.43	5.55	3.72
Total Lys, %	1.54	1.48	1.68
Ca, %	0.65	0.75	0.69
P, %	0.64	0.62	0.68
Available P, %	0.55	0.47	0.38

A total of 360 weanling pigs (DNA 241 × 600) were used in a three-phase nursery trial with six pigs per pen and 10 replicates per treatment. Treatment diets were fed from days 0 to 7 (Phase 1) and days 7 to 19 (Phase 2). A common diet was fed from days 19 to 35 (Phase 3).

^*a*^Provided per kilogram of premix: 22 g Mn from manganese oxide; 73 g Fe from iron sulfate; 73 g Zn from zinc sulfate; 11 g Cu from copper sulfate; 198 mg I from calcium iodate; and 198 mg Se from sodium selenite.

^*b*^Provided per kilogram of premix: 3,527,360 IU vitamin A; 881,840 IU vitamin D3; 17,637 IU vitamin E; 3,307 mg riboflavin; 1,764 mg menadione; 11,023 mg pantothenic acid; 33,069 mg niacin; and 15.4 mg vitamin B12.

^*c*^Ronozyme HiPhos 2700, DSM Nutritional Products, Parsippany, NJ.

^*d*^Hamlet Protein, Findley, OH.

^*e*^Diets included either 1.5% choice white grease (control); 1.5% choice white grease plus 3,000 ppm ZnO in phase 1 or 2,000 ZnO in phase 2; 1.5% choice white grease plus 50 g/d Carbadox (Phibro Animal Health, Teaneck, NJ); 0.5% choice white grease plus 1.0% C6:0, C8:0, and C10:0 in a 1:1:1 blend; 0.5% choice white grease plus 1% Feed Energy proprietary vegetable oil blend (Feed Energy Company, Des Moines, IA); or 0.5% choice white grease plus 1% FORMI GML (ADDCON GmbH, Bitterfeld-Wolfen, Germany).

During the entire treatment period (days 0 to 19), pigs fed diets containing ZnO or carbadox had greater (*P* < 0.05) ADG than those fed control diets or diets containing the Feed Energy Proprietary Oil Blend. Other controlled research has demonstrated that the use of antibiotics, like carbadox, results in increased growth performance. In fact, Puls et al. (2018) found similar results when feeding two antibiotic feeding programs, one of which consisted of carbadox, and comparing them to nonmedicated control diets. Their results also displayed an increase in ADG, but no significant effect on feed efficiency. However, in the current experiment, there was a substantial feed intake improvement (*P* < 0.05) in pigs fed diets containing ZnO compared with those fed control diets or the MCFA or Feed Energy Proprietary Oil Blends, but there was no overall difference in feed efficiency during the treatment period.

As expected, there were no discernable differences (*P* > 0.10) in pigs fed common diets during phase 3 (days 19 to 35). However, there was sufficient difference in early growth performance to cause significant differences in both ADG and ADFI overall (days 0 to 35). Although all treatments had pigs starting with the same average weight, up to 0.32-kg difference in body weight was observed among treatments just 1-wk postweaning. By the end of the 35-d experiment, pigs fed diets containing ZnO or carbadox were at least 1.05 or 0.73 kg heavier than those fed control diets or diets containing the MCFA or Feed Energy Proprietary Oil Blends.

This study shows that ZnO and carbadox are valuable additives to help maximize performance in the early nursery period. These findings coincide with other research that states ZnO can promote growth performance during the postweaning period when included at pharmacological levels (Sales, 2013). However, this research also demonstrates that some lipid-containing feed additives, such as FORMI GML, may result in similar performance as ZnO and feed-based antibiotics. Yet, it was also determined that other MCFA products may actually reduce feed intake and subsequent growth when included in early nursery diets. Thus, when comparing the results of this study to others within this field, findings are variable. The current experiment showed that the FORMI GML product can yield similar growth performance as ZnO and carbadox, but it is unknown what specific mode of action allowed this product to perform in such a way. One possibility could be the specific MCFA profile in FORMI GML. The analyzed feed samples suggest that there was some variation in levels of MCFA in each diet, which could have affected the efficacy of each product in this scenario. Controlled research by Gebhardt et al. (2017) described that a blend of MCFA in nursery pig diets can result in improvement in growth performance; however, the effects of MCFA depend on the type and inclusion rate. Improvements in nursery pig growth performance were observed by Gebhardt et al. (2017) by including 0.50% C6 or C8 with 0.25 to 1.50% of a 1:1:1 blend of C6, C8, and C10. Therefore, further research is needed to study specific MCFA concentrations and how they affect growth performance at different inclusion levels (Table 2).

**Table 2.** Chemical analysis of experimental diets

Item;	Control	3,000 ppm P1 2,000 ppm P2 ZnO	50 g/ton Carbadox	C6:C8:C10 Blend	Proprietary Oil Blend by Feed Energy	FORMI GML by ADDCON
Phase 1						
Dry matter, %	88.4	88.6	89.1	88.6	89.2	88.6
Crude protein, %	20.8	21.0	21.2	20.8	21.1	20.8
P, %	0.70	0.67	0.71	0.72	0.71	0.70
Ca, %	0.97	0.90	1.17	1.00	0.97	0.96
C6:0	<0.01	<0.01	<0.01	<0.01	<0.01	<0.01
C8:0	<0.01	<0.01	<0.01	0.05	0.05	<0.01
C10:0	<0.01	<0.01	<0.01	0.10	0.10	0.01
C12:0	<0.01	<0.01	<0.01	0.05	0.05	0.10
Phase 2						
Dry matter, %	86.9	87.0	87.3	86.7	87.4	86.8
Crude protein, %	21.3	20.5	21.6	21.1	21.8	22.3
P, %	0.66	0.6	0.65	0.65	0.71	0.67
Ca, %	1.25	1.04	1.54	1.15	1.27	0.99
C6:0	<0.01	<0.01	<0.01	<0.01	<0.01	<0.01
C8:0	<0.01	<0.01	<0.01	0.07	<0.01	0.01
C10:0	<0.01	<0.01	<0.01	0.12	<0.01	0.02
C12:0	<0.01	<0.01	<0.01	<0.01	<0.01	0.09
Phase 3						
Dry matter, %	88.3	–	–	–	–	–
Crude protein, %	21.5	–	–	–	–	–
P, %	0.63	–	–	–	–	–
Ca, %	1.08	–	–	–	–	–
C6:0	<0.01	–	–	–	–	–
C8:0	<0.01	–	–	–	–	–
C10:0	<0.01	–	–	–	–	–
C12:0	<0.01	–	–	–	–	–

Complete diet samples were obtained from each dietary treatment and the common phase 3 diet during daily feed additions, representing at least 10 different samples per diet. Samples of diets were pooled and analyzed for DM, CP, P, Ca, and medium-chain fatty acids (Midwest Laboratories Inc., Omaha, NE).

### Blood Parameters

Day 0 blood data were collected and analyzed as a baseline for comparison. Although discrepancies were detected (*P* = 0.0351) for day 0 bicarbonate concentrations, by day 21 these values became similar (*P* = 0.0372). On day 21, pigs fed ZnO or carbadox had higher (*P* < 0.0001) glucose values than those fed the Feed Energy Proprietary Oil Blend, with all other treatments intermediate. Pigs fed carbadox had higher (*P* = 0.0002) total calcium than the negative control, MCFA blend, Feed Energy Proprietary Oil Blend, or FORMI GML diets, with ZnO being intermediate. Sodium–potassium ratio was higher (*P* = 0.0354) for pigs fed ZnO than carbadox, with all other diets intermediate. On day 21, aspartate transaminase concentrations were greater (*P* = 0.043) for pigs fed ZnO than those fed MCFA blend, with all other treatments being intermediate. Similarly, the ZnO treatment resulted in higher (*P* < 0.0001) alkaline phosphatase concentrations compared with all other treatments. Differences in day 21 urea nitrogen and anion gap were marginally significant (*P* = 0.056 and *P* = 0.070, respectively). No significant impact (*P* > 0.10) was found for day 21 concentrations of creatinine, protein, albumin, globulin, phosphorus, sodium, potassium, chloride, sorbitol dehydrogenase, creatine kinase, or bilirubin. These findings indicate that carbadox may provide a health benefit to pigs. Although the ZnO diet proved higher blood glucose values, it may also contribute to hepatic issues. Other diets remain intermediate. Further research is necessary to better comprehend the effects of MCFA on blood serum chemistry and hepatic profiles (Table 3).

**Table 3.** Effects of ZnO, carbadox, or various lipid additives on nursery pig growth performance

	3,000 ppm P1 2,000 ppm P2 ZnO	Control	50 g/ton Carbadox	C6, C8, C10 Blend	Proprietary Oil blend by Feed Energy	FORMI GML by ADDCON	SEM	*P*
BW, kg								
d 0 (weaning)	5.42	5.42	5.41	5.42	5.41	5.42	0.009	0.696
d 7	6.10^a^	6.02^ab^	6.13^a^	5.79^b^	5.82^ab^	6.11^a^	0.075	0.003
d 19	10.26^a^	9.23^c^	10.05^ab^	9.34^bc^	9.11^c^	9.71^abc^	0.170	< 0.0001
d 35	18.70	17.66	18.49	17.84	17.47	17.99	0.316	0.06
Phase 1 (d 0 to 7)								
ADG, g/d	97^abc^	85^abc^	103^a^	52^c^	59^bc^	98^ab^	10.7	0.003
ADFI, g/d	137^a^	116^abc^	117^ab^	92^bc^	85^bc^	119^ab^	7.3	< 0.0001
G:F	0.68	0.72	0.87	0.57	0.66	0.81	0.071	0.055
Phase 2 (d 8 to 19)								
ADG, g/d	347^a^	270^c^	325^ab^	297^bc^	278^bc^	300^abc^	11.4	0.0001
ADFI, g/d	428^a^	370^ab^	407^ab^	361^b^	355^b^	381^ab^	14.2	0.004
G:F	0.81^ab^	0.74^b^	0.80^ab^	0.81^a^	0.79^ab^	0.79^ab^	0.019	0.043
Overall Treatment (d 0 to 19)								
ADG, g/d	255^a^	202^c^	242^ab^	207^bc^	197^c^	226^abc^	8.7	< 0.0001
ADFI, g/d	321^a^	276^b^	298^ab^	262^b^	255^b^	284^ab^	10.6	0.0004
G:F	0.79	0.73	0.81	0.79	0.77	0.80	0.018	0.078
Common Phase 3 (d 20 to 35)								
ADG, g/d	523	516	533	531	517	518	12.6	0.873
ADFI, g/d	793	756	781	757	723	743	17.9	0.089
G:F	0.66	0.69	0.68	0.70	0.72	0.70	0.015	0.158
Overall (d 0 to 35)								
ADG, g/d	377^a^	344^ab^	374^ab^	355^ab^	339^b^	359^ab^	8.5	0.012
ADFI, g/d	536^a^	492^ab^	517^a^	488^ab^	463^b^	494^ab^	11.5	0.001
G:F	0.70	0.70	0.72	0.73	0.73	0.73	0.012	0.32

A total of 360 weanling pigs (6 pigs per pen, 10 pens/treatment) were fed treatment diets during phase 1 (days 0 to 7) and phase 2 (days 8 to d 19). A common diet was fed from days 20 to 35.

^abc^Means within a row that do not share a common superscript differ *P* < 0.05.

### Fecal Consistency and Gut Microflora

Initial fecal scoring on day 0 of the experiment showed similar fecal scores for all pigs at placement. However, on days 1, 2, 7, 14, and 19, pigs fed the ZnO and carbadox treatment had significantly lower fecal scores (*P* < 0.05) when compared with those being fed the control diet or diets containing MCFA blend, Feed Energy Proprietary Oil Blend, or FORMI GML. Similarly, findings from Cochrane et al. (2018) and Mohana Devi and Kim, (2014) also showed minimal impact of MCFA supplementation on fecal score. Although this study demonstrated the ability of FORMI GML to result in similar growth performance as ZnO and carbadox, it did not have the same impact on fecal consistency, as piglets being fed the product had softer feces. Finally, upon transitioning pigs to the phase 3 common diet on day 21, fecal scores standardized across treatment.

Fecal samples were collected on day 0 to determine baseline microflora. All 36 pigs sampled at day 0 were positive at various increments for *Clostridium perfringens.* Across *E. coli* strains, 28 pigs tested positive, with three pigs showing moderate to high *E. coli hemolytic* growth. Eleven pigs were positive for *Enterococcus spp.* Of particular interest, two pigs were positive for *Streptococcus suis.* On day 21, microflora were greatly reduced with no particular differences across treatments. Of 33 samples, 25 showed no significant microbial growth. Only two species were present in detectable levels—two samples showed moderate to high growth of Lactose-hemolytic *E. coli*, and six displayed high growth of hemolytic *E. coli*. Three hemolytic *E. coli* cultures from the day 0 fecal swabs and one culture from day 21 swabs were analyzed for antimicrobial resistance. All three day 0 cultures showed resistance to clindamycin, penicillin, tiamulin, and tilmicosin. Two samples showed resistance to ampicillin, sulfadimethocine, tetracycline, and trimethoprim. The day 21 culture showed resistance to clindamycin, penicillin, sulfadimethoxine, tetracycline, tiamulin, tilmicosin, and neomycin. Ultimately, diet had no significant impact on nursery pig gut microbiota or antibiotic resistance among bacterial strains. Research should be continued with greater replication to further evaluate the effects of MCFA on gut microflora and fecal consistency.
